# A Comparison of Users and Nonusers of a Web-Based Intervention for Carers of Older Persons With Alzheimer Disease and Related Dementias: Mixed Methods Secondary Analysis

**DOI:** 10.2196/14254

**Published:** 2019-10-17

**Authors:** Wendy Duggleby, Jenny Ploeg, Carrie McAiney, Kathryn Fisher, Kathya Jovel Ruiz, Sunita Ghosh, Shelley Peacock, Maureen Markle-Reid, Allison Williams, Jean Triscott, Jennifer Swindle

**Affiliations:** 1 Faculty of Nursing University of Alberta Edmonton, AB Canada; 2 School of Nursing Faculty of Health Sciences McMaster University Hamilton, ON Canada; 3 School of Public Health and Health Systems University of Waterloo Waterloo, ON Canada; 4 Cancer Care Alberta Health Services Edmonton, AB Canada; 5 College of Nursing University of Saskatchewan Saskatoon, SK Canada; 6 School of Geography and Earth Sciences McMaster University Hamilton, ON Canada; 7 Faculty of Medicine and Dentistry University of Alberta Edmonton, AB Canada

**Keywords:** Web-based intervention, carers, dementia, multiple chronic conditions, program evaluation

## Abstract

**Background:**

A self-administered Web-based intervention was developed to help carers of persons with Alzheimer disease and related dementias (ADRD) and multiple chronic conditions (MCC) deal with the significant transitions they experience. The intervention, My Tools 4 Care (MT4C), was evaluated during a pragmatic mixed methods randomized controlled trial with 199 carers. Those in the intervention group received free, password-protected access to MT4C for three months. MT4C was found to increase hope in participants at three months compared with the control group. However, in the intervention group, 22% (20/92) did not use MT4C at all during the three-month period.

**Objective:**

This mixed methods secondary analysis aimed to (1) examine differences at three months in the outcomes of hope, self-efficacy, and health-related quality of life (HRQOL) scores in users (ie, those who used MT4C at least once during the three-month period) compared with nonusers and (2) identify reasons for nonuse.

**Methods:**

Data from the treatment group of a pragmatic mixed methods randomized controlled trial were used. Through audiotaped telephone interviews, trained research assistants collected data on participants’ hope (Herth Hope Index; HHI), self-efficacy (General Self-Efficacy Scale; GSES), and HRQOL (Short-Form 12-item health survey version 2; SF-12v2) at baseline, one month, and three months. Treatment group participants also provided feedback on MT4C through qualitative telephone interviews at one month and three months. Analysis of covariance was used to determine differences at three months, and generalized estimating equations were used to determine significant differences in HHI, GSES, and SF-12v2 between users and nonusers of MT4C from baseline to three months. Interview data were analyzed using content analysis and integrated with quantitative data at the result stage.

**Results:**

Of the 101 participants at baseline, 9 (9%) withdrew from the study, leaving 92 participants at three months of which 72 (78%) used MT4C at least once; 20 (22%) participants did not use it at all. At baseline, there were no statistically significant differences in demographic characteristics and in outcome variables (HHI, GSES, and SF-12v2 mental component score and physical component score) between users and nonusers. At three months, participants who used MT4C at least once during the three-month period (users) reported higher mean GSES scores (*P*=.003) than nonusers. Over time, users had significantly higher GSES scores than nonusers (*P*=.048). Reasons for nonuse of MT4C included the following: caregiving demands, problems accessing MT4C (poor connectivity, computer literacy, and navigation of MT4C), and preferences (for paper format or face-to-face interaction).

**Conclusions:**

Web-based interventions, such as MT4C, have the potential to increase the self-efficacy of carers of persons with ADRD and MCC. Future research with MT4C should consider including educational programs for computer literacy and providing alternate ways to access MT4C in addition to Web-based access.

**Trial Registration:**

ClinicalTrials.gov NCT02428387; https://clinicaltrials.gov/ct2/show/NCT02428387

## Introduction

Family carers (unpaid family or friends) of persons with Alzheimer disease and related dementias (ADRD) have been recognized worldwide as providers of the majority of care [1]. The need to support these family carers is well documented as they experience significant changes in their lives [2] that can negatively impact their physical and mental health [3,4]. Family carers have been found to seek information for themselves and others using computers, smartphones, or other electronic means more frequently than noncarers [5]. Web-based interventions to support family carers are increasingly becoming available and affordable, flexible, and accessible [6]. In a recent meta-analysis, Web-based interventions for family carers were found to have positive outcomes such as increased mental health [7] and self-efficacy [8]. However, carers have also reported barriers to using Web-based interventions such as difficulty with language and computer literacy [9] and with navigation of the websites [10]. The limited interaction with other carers when using Web-based interventions has also been a concern [11,12].

A Web-based intervention, My Tools 4 Care (MT4C), was developed to support family carers of persons with ADRD and multiple chronic conditions (MCC). MT4C was initially developed as a hard copy workbook that was piloted and showed promise in terms of helping family carers of persons with dementia [13]. The next step was to work with Atmist (Web developers), the research team, and family carers to develop a feasible, acceptable, and easy-to-use Web-based version. MT4C [14] is a self-administered, flexible, tailored intervention as carers decide which activities they wish to engage in and when. It can be used on a computer, tablet, or smartphone. MT4C was evaluated during a pragmatic mixed methods randomized control trial with 199 carers between June 2015 and April 2017 and was found to significantly increase participants’ hope in the treatment group compared with the control group [15].

Of 92 participants in the treatment group, 20 (22%) did not use MT4C over the three-month period. This is similar to other Web-based internet intervention studies that reported a substantial number of treatment group participants who did not use the interventions [10,12]. The Consolidated Standards of Reporting Trials-Electronic Health guidelines for reporting Web-based intervention trials recommend the common practice of using an intent-to-treat analysis in trials that include users and nonusers [16]. However, it is also important to examine the data of nonuser participants [17]. This paper reports a secondary analysis to provide insight into the characteristics and the difference in outcomes of hope, general self-efficacy, and quality of life in users versus nonusers of MT4C. This examination of MT4C will inform the evaluation of future Web-based interventions for family carers of persons with ADRD and MCC.

The aim of this secondary analysis was to examine differences in outcomes (hope, self-efficacy, and quality of life) in participants who used MT4C (users) in a three-month period versus those who did not use it (nonusers) and to examine reasons for nonuse. The following research questions guided the study:

1. Was there a significant difference in demographic characteristics in the users versus nonusers?
2. Was there a significant difference in hope, self-efficacy, and quality of life at three months in users versus nonusers?
3. What were the reasons for nonuse from the qualitative data collected for nonusers?

Those who did not use MT4C were not a control group, as they had the opportunity to use it. The nonusers had been randomly assigned to the treatment group but chose not to use MT4C. We hypothesized that the users of MT4C will report a statistically significant increase in hope, self-efficacy, and quality of life compared with the nonusers.

## Methods

### Design

A detailed protocol [18] and 2 articles describing MT4C and its evaluation have been published elsewhere [15,19]; thus, only details relevant to this secondary analysis are provided herein. Similar to the pragmatic trial, this study utilized a mixed methods comparative design. The data reported here focus on family carers allocated to the treatment group (N=101). Qualitative data from the interviews and quantitative data from the treatment group collected during the original study were integrated in the results stage. Qualitative data were used to understand the quantitative results.

### Ethics

The primary study received ethical approval from the University of Alberta Health Research Ethics Board (No. Pro00048721) and the Hamilton Integrated Research Ethics Board (No. 15-309). The initial ethics application included the ability to conduct a secondary analysis of the data.

### Recruitment of Participants

Family carers were invited to participate if they were over the age of 18 years and were caring for a person aged 65 years or older living with ADRD and MCC in the community. In addition, they needed to have a valid email address and access to a computer. Family carers were recruited through multiple community organizations including Alzheimer Society branches in each province and advertisements in local community newspapers in Alberta. If they met the eligibility criteria, they were asked to contact the researchers. Participants were randomly assigned to a treatment or control group using stratified permuted block randomization. Different consent forms (one for the treatment group and one for the control group) were used to blind participants regarding their group assignment.

**Figure 1:**
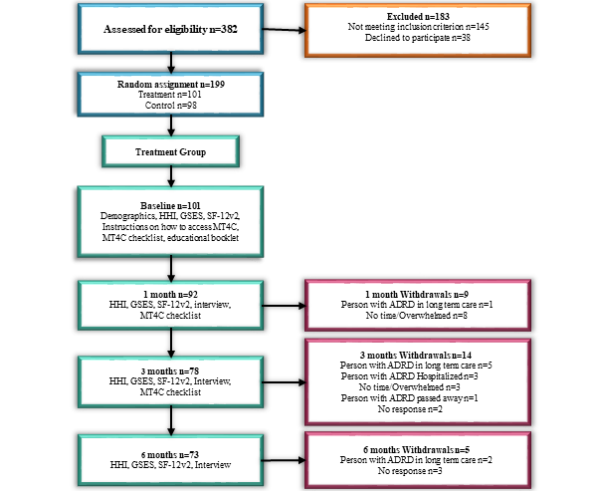
Data collection procedures and numbers of participants. ADRD: Alzheimer disease and related dementias; GSES: general self-efficacy scale; HHI: herth hope index; MT4C: My Tools 4 Care; SF-12v2: short-form 12-item health survey.

Once consent was obtained, trained data collectors collected all data (baseline, one month, and three months) via telephone and entered it into REDCap, a secure, password-protected Web-based data collection service, offered at the University of Alberta. Data collection occurred from June 2015 to April 2017 and is reported in more detail in the study protocol [18]. Data collection procedures for the treatment group and the number of participants at each period are presented in Figure 1.

### Intervention

Following baseline interviews, participants in the treatment group received free, password-protected access to MT4C for three months. Research assistants, using a standardized script, instructed the participants to access MT4C at their convenience on a computer, tablet, or smartphone. A follow-up email was sent to participants in the treatment group with instructions on how to access the site and their login information. After logging in, the first page provided instructions on how to use MT4C. Each Web page also contained a menu outlining the sections that comprised MT4C: (1) about me; (2) common changes to expect; (3) frequently asked questions; (4) resources; (5) important health information; and (6) calendar. MT4C also provided options to add formatted text, photos, and PDF files in certain sections. All information entered by participants was treated confidentially and was not accessible to the study team. Participants also received an electronic copy of the Alzheimer Society’s *The Progression of Alzheimer’s Disease* booklet [20], a copy of the study questionnaires, and the MT4C toolkit checklist intended for participants to record their use of the MT4C site. During the one-month interview, trained data collectors encouraged nonusers to use MT4C. No changes or alterations were made to MT4C during the study.

### Measures

#### Data Collection

Data collected at baseline included age, gender, years in caregiver role, employment status, ethnicity, household income before taxes, living arrangement, the relationship to the person with ADRD and MCC, and any assistance with caregiving. Data regarding sex, age, and number of chronic conditions of the person with ADRD and MCC were also collected.

Data on family carers’ hope, self-efficacy, and health-related quality of life (HRQOL) were collected at baseline, one month, three months, and 6 months by trained research assistants using the measures outlined in the following section. Figure 1 outlines the data collection procedures.

#### Herth Hope Index

To measure hope using the Herth Hope Index (HHI), participants answered 12 questions scored on a Likert-type scale from 1 (strongly disagree) to 4 (strongly agree). A total hope score (range 12-48) was reported (higher scores indicate higher hope) along with 3 subscales: (1) temporality and future, (2) positive readiness and expectancy, and (3) interconnectedness. The HHI has a test-retest reliability of 0.91 (*P*<.05) and criterion-related validity r of 0.81 to 0.92 (*P*<.005) [21].

#### General Self-Efficacy Scale

The General Self-Efficacy Scale (GSES) is a measure of perceived self-efficacy or belief that one can deal with difficult tasks or cope with adversity using a 10-item 4-point scale [22]. Total scores ranged from 10 to 40. It is a reliable tool with a Cronbach alpha coefficient ranging from .76 to .90 (*P*<.05).

#### Short Form 12-Item Health Survey

The SF-12v2 is a measure of HRQOL, consisting of 12 questions measuring 8 domains of well-being and functioning (physical functioning, role functioning, bodily pain, general health, vitality, social functioning, emotional health, and mental health) [23,24]. Responses to the 12 questions are summarized by 2 scores: physical component summary score (PCS; estimated test-retest reliability of *r*=0.89) and a mental component summary score (MCS; estimated test-retest reliability *r*=0.86) that range from 0 to 100 [25].

#### Qualitative Interviews

Interviews were semistructured and completed over the telephone by a trained research assistant. All interviews were audio-recorded and transcribed verbatim by an experienced transcriptionist. Participants were asked questions such as *What were you thinking about when you worked on MT4C?*; *Did it help you deal with significant changes?*; *What did you like best?*; and *What did you like least?* As indicated by the larger study protocol, qualitative interviews were conducted with a subsample of study participants. For those in the treatment group, 6 of the 20 nonusers were interviewed using semistructured interviews.

#### My Tools 4 Care Checklist (Use of My Tools 4 Care)

The MT4C checklist was developed by the research team and was used to collect data on the participants’ use of MT4C. The checklist was intended for participants to keep track of the number of times they accessed each section of MT4C and the amount of time spent on each section. Data from the checklist were used to determine the use of MT4C at one month and three months.

Participants also made comments on the checklist about their nonuse, which were considered qualitative data for this study.

### Data Analysis

Data were entered in SPSS version 24 (IBM) and checked for accuracy by a trained research assistant. Before data analysis, participants were divided into 2 groups: (1) participants who used MT4C at least once over three months and (2) participants who did not use MT4C within the three-month period. Use of MT4C was captured using a dichotomous variable, where 1=used MT4C at least once during the three-month intervention period and 0=did not use MT4C during the three-month intervention period.

#### Participant Characteristics

Means and SDs were used to represent continuous demographic characteristics of participants and persons with ADRD and MCC; categorical data were reported with numbers and percentages. Chi-squared statistical analysis and *t* tests were used to determine differences in demographic characteristics between the groups.

#### Outcome Measures

Analysis of covariance (ANCOVA) was used to test the differences in outcome variables between users and nonusers at three months. Separate ANCOVA models were run for each outcome, with the three-month outcome as the dependent variable, group (users and nonusers) as the independent variable, and baseline value of the outcome as the covariate. A *P* value of <.05 was used for statistical significance, and 2-sided tests were used. A complete case analysis was used, which means we did not impute for missing data (ie, we used people who had a complete record for the 3 time points baseline, one month, and three months).

Generalized estimating equations (GEE) were used to determine differences between the 2 groups (users vs nonusers) over time for the main outcome variables of HHI, GSES, and SF-12v2 MCS and PCS. GEE is an alternative statistical method appropriate for repeated measures data and is more flexible than other methods (eg, repeated measures ANCOVA) because it does not require that outcomes be normally distributed and can handle both continuous and dichotomous outcomes. It can also be used with small sample sizes [26]. Use was captured dichotomously at 3 time points in the GEE models, with use=0 for all intervention group participants at baseline, and 1 (used) or 0 (did not use) at one month and three months (depending on use reported by participants at these time points). Separate GEE models were run for each outcome (primary and secondary).

#### Reasons for Nonuse of My Tools 4 Care

Nonusers’ MT4C checklist and qualitative data from interviews were analyzed using content analysis [27] and informed the quantitative data in the results phase. Transcripts were read overall by a trained research assistant and organized into categories to address the study purpose. Trustworthiness of the data was maintained by keeping an audit trail and using participants’ words as much as possible.

## Results

### Comparison of User and Nonuser Participants

A total of 101 participants were allocated to the treatment group at baseline. Following baseline measures, 9 participants withdrew and the remaining 92 participants received instructions on how to access MT4C. Figure 1 illustrates the number of persons at baseline and three months. The mean age of all participants in the treatment group was 63.5 years (SD 12.0), and they had been carers for an average of 4.1 years (SD 3.9). The majority of participants were female (73/92, 79%), white (84/92, 91%), were living with a person with ADRD (63/92, 62%), and were the spouse of a person with ADRD (48/92, 52%). No statistically significant differences were found in the demographic characteristics of users and nonusers (Table 1).

The means and SDs of the outcome measures (HHI, GSES, MCS, and PCS for each group at baseline and three months) are presented in Table 2.

Table 3 provides the ANCOVA results for each outcome. The group variable (users and nonusers) was significant for the GSES outcome (*P*=.003), indicating that the use of MT4C during the three-month period was associated with an increase in GSES from baseline to three months. The use of MT4C was not associated with significant differences in the other outcomes.

Table 4 provides the GEE model results and shows that the use of MT4C was associated with an increase in GSES over three months (*P*=.048). The use of MT4C was not associated with significant changes in the other outcomes.

**Table 1:** My Tools 4 Care users versus nonusers: baseline comparison characteristics.

Characteristics			Used MT4C^a^ (N=72)	Did not use MT4C (N=20)	Total sample (N=92)	*P* value
**Carers**						
	**Gender, n (%)**					
		Male	14 (19)	5 (25)	19 (20)	.59
		Female	58 (80)	15 (75)	73 (79)	.59
	Age (years), mean (SD)		62.8 (12.2)	65.8 (11.3)	63.5 (12.0)	.38
	Caregiving (years), mean (SD)		3.9 (4.0)	4.8 (3.6)	4.1 (3.9)	.38
	Education (years), mean (SD)		14.2 (2.9)	14.2 (2.8)	14.2 (2.9)	.10
	Chronic conditions, mean (SD)		2.3 (1.6)	2.2 (1.3)	2.2 (1.6)	.86
	**Marital status, n (%)**					
		Married or living with someone	60 (83)	17 (85)	77 (84)	.87
		Single, widowed, divorced/separated	12 (17)	3 (15)	15 (16)	.87
	**Ethnicity, n (%)**					
		White	66 (9)	18 (90)	84 (9)	.97
		Other	5 (7)	2 (10)	7 (8)	.97
	**Employed, n (%)**					
		Yes	31 (4)	3 (15)	34 (3)	.05
		No	41 (57)	16 (84)	57 (6)	.05
	**Living with care recipient, n (%)**					
		Yes	50 (6)	13 (6)	63 (68)	.71
		No	22 (31)	7(35)	29 (31)	.71
	**Relationship to care recipient, n (%)**					
		Husband/wife/life partner	37 (51)	11 (55)	48 (52)	.77
		Other	35 (9)	9 (45.0)	44 (48)	.77
	**Finances meet needs, n (%)**					
		Completely, very well, adequately	60 (8)	14 (7)	74 (80)	.18
		With some difficulty, not very well, totally inadequate	12 (17)	6 (30)	18 (19)	.18
	**Household income, n (%)**					
		<40,000	17 (24)	5 (25)	22 (24)	.99
		>40,000 and <70,000	18 (25)	4 (20)	22 (24)	.99
		>70,000	26 (36)	8 (40)	34 (37)	.99
		No response	11 (15)	3 (15)	14 (15)	.99
	**Assistance with caring, n (%)**					
		Yes	49 (68)	15 (75)	64 (70)	.55
		No	23 (32)	5 (25)	28 (30)	.55
**Care recipient**						
	**Gender, mean (%)**					
		Male	37 (51)	11 (55)	48 (52)	.77
		Female	35 (49)	9 (45)	44 (48)	.77
	Age (years), mean (SD)		79.6 (7.7)	82.5 (6.5)	80.2 (7.5)	.13
	Chronic conditions, mean (SD)		10.6 (4.2)	10.1 (4.1)	10.5 (4.2)	.63

**^a^**MT4C: My Tools 4 Care.

**Table 2:** Mean and SD of outcomes at baseline and three months for users and nonusers.

Outcomes		Users MT4C^a^ (N=72), mean (SD)	Nonusers MT4C (N=20), mean (SD)
**Outcomes at baseline**			
	PCS^b^	50.80 (12.01)	50.51 (9.62)
	MCS^c^	46.41(10.38)	44.19 (11.66)
	HHI^d^	39.08 (4.72)	37.78 (6.16)
	GSES^e^	32.41 (4.15)	31.37 (4.17)
**Outcomes at one month**			
	PCS	50.74 (11.14)	48.20 (8.83)
	MCS	47.57 (10.26)	45.83 (12.89)
	HHI	39.45 (5.07)	38.61 (5.92)
	GSES	32.99 (4.01)	30.02 (4.92)
**Outcomes at three months**			
	PCS	50.25 (10.99)	50.37 (7.84)
	MCS	47.48 (9.74)	44.69 (12.02)
	HHI	39.89 (5.18)	38.52 (5.70)
	GSES	32.76 (4.43)	29.21(6.19)

^a^MT4C: My Tools 4 Care.

^b^PCS: physical component score (SF-12v2).

^c^MCS: mental component score (SF-12v2).

^d^HHI: herth hope index.

^e^GSES: general self-efficacy scale.

**Table 3:** Analysis of covariance results for outcomes from baseline to three months for users versus nonusers (group).

Outcome			Parameter estimate (95% CI)	*P* value
**SF-12v2^a^ (PCS^b^ and MCS^c^; n=76)**				
	**PCS at three months**			
		Intercept	16.09 (8.60 to 23.58)	<.001
		PCS—baseline	0.70 (0.56 to 0.83)	<.001
		Group	−1.36 (−5.43 to 2.72)	.51
	**MCS at three months**			
		Intercept	16.77 (6.03 to 27.51)	.003
		MCS at baseline	0.59 (0.39 to 0.79)	<.001
		Group	2.79 (−2.35 to 7.93)	.28
**HHI^d^(n=78)**				
	**HHI factor 1 at three months**			
		Intercept	2.16 (−0.48 to 4.80)	.11
		HHI factor 1 at baseline	0.81 (0.61 to 1.00)	<.001
		Group	0.59 (−0.35 to 1.53)	.22
	**HHI factor 2 at three months**			
		Intercept	4.21 (1.75 to 6.67)	.001
		HHI factor 2 at baseline	0.67 (0.49 to 0.84)	<.001
		Group	0.49 (−0.29 to 1.26)	.22
	**HHI factor 3 at three months**			
		Intercept	4.62 (2.09 to 7.15)	<.001
		HHI factor 3 at baseline	0.62 (0.43 to 0.81)	<.001
		Group	0.49 (−0.40 to 1.38)	.28
	**HHI total score at three months**			
		Intercept	5.50 (−1.62 to 12.62)	.13
		HHI at baseline	0.84 (0.66 to 1.01)	<.001
		Group	1.47 (−0.62 to 3.56)	.17
**GSES^e^ (n=77) at three months**				
		Intercept	4.14 (−2.59 to 10.86)	.22
		GSES at baseline	0.78 (0.58 to 0.98)	<.001
		Group	3.23 (1.12 to 5.33)	.003^f^

^a^SF-12v2: short-form 12-item health survey.

^b^PCS: physical component score (SF-12v2).

^c^MCS: mental component score (SF-12v2).

^d^HHI: herth hope index.

^e^GSES: general self-efficacy scale.

^f^Significant at *P<*.05.

**Table 4:** Generalized estimating equation results for outcomes (repeated measures analysis over three months [time 2=one month; time 3=three months]) for users compared with nonusers. Time 2 (one month from baseline) was not a significant factor in time 3 outcomes.

Outcome				Estimate	SE (95% CI)	*P* value
**SF-12v2^a^**						
	**PCS^b^**					
		**Time 3^c^**				
			Group (users)	−2.03	1.68 (−5.32 to 1.27)	.23
			Time 2	−0.23	0.94 (−2.07 to 1.60)	.80
	**MCS^d^**					
		**Time 3^c^**				
			Group (users)	0.89	1.70 (−2.44 to 4.21)	.60
			Time 2	−0.15	1.06 (−2.22 to 1.92)	.89
**HHI^e^ total**						
		**Time 3^c^**				
			Group (users)	−0.38	0.86 (−2.06 to 1.30)	.66
			Time 2	−0.44	0.52 (−1.45 to 0.58)	.40
**GSES^f^**						
		**Time 3^c^**				
			Group (users)	1.55	0.78 (0.01 to 3.09)	.048^g^
			Time 2	0.06	0.42 (−0.77 to 0.89)	.89

^a^SF-12v2: short-form 12-item health survey.

^b^PCS: physical component score (SF-12v2).

^c^Reference group.

^d^MCS: mental component score (SF-12v2).

^e^HHI: herth hope index.

^f^GSES: general self-efficacy scale.

^g^Significant at *P*<.05.

### Reasons for Nonuse of My Tools 4 Care

Reasons for nonuse of MT4C reported in the qualitative data included the following: caregiving demands; problems accessing and navigating the site; and preference for paper or in-person contact. Participants reported being consumed with the role of caregiving and as a result did not have enough time to use MT4C. As one participant said: “...and I got to admit that it was, uh, something that, uh, I didn’t go onto too much, just strictly because of all the other things that were—were going on this past month.” Those who were able to find a little bit of time to look at MT4C found they were quickly distracted by the care recipient and ultimately did not use it. One participant described having to stop using MT4C to tend to her husband: “Well, um, I just finished reading it, and—and—and, then, I had to go off because I had to go help my husband.”

Caregiving demands also resulted in nonuser participants feeling stressed. As one participant said: “I’m extremely stressed with taking care of my wife, and so I lost the email with login instructions.” Another indicated that the lack of energy was a factor “...[I] work full time early morning to late evening...and at the end of the day, I don’t have the energy or time to go on the computer.”

Problems accessing MT4C were related to poor internet connections, computer literacy, and difficulties navigating the site. Nonusers who lived in rural areas reported poor internet connections: “...my internet connection at home is poor—I live in a rural area.” Several nonusers described their lack of experience with computers (computer literacy): “No, it—it’s, uh, as far as the computer is concerned, it’s the—the operator of it that’s at fault.”; another said: “Um, well, I get frustrated at myself when, you know, I’m working on the website...”

In terms of difficulty navigating the site, participants described forgetting the link to log in and difficulty printing instructions for the site. A participant described her frustration with not being able to find where she had previously been working on the site after being interrupted by her husband, “he kept interrupting me. Then, I couldn’t find where I left off to continue...” Caregiving demands coupled with navigating the site were the reason this participant did not use MT4C.

Nonusers also seemed to have a preference for access to hard copy or paper format of MT4C and interaction with other carers. As a participant said, “...Sometimes, you actually have to have something printed in front of you, uh, and I’m better off—I’m better with paper. In some instances, to sit and reflect, I’m not really good at what—I’m not really one of those people who can do it all on-line.” This participant described his lack of experience with working on the Web, but also suggested his preference was for paper. Another participant suggested a preference for social interaction rather than Web-based tools: “I think—I think I know—and this is [chuckles]—this isn’t specific to this Toolkit, but it sort of relates to it: um, I think I’m the kind of person who gets a lot more out of, you know, actual social interaction around something.”

The reasons for nonuse of MT4C, although reported as separate issues, appeared to be interrelated. For example, the lack of computer literacy meant it took more time to use MT4C, but with caregiving demands, less time was available. Furthermore, participants who are not computer literate became frustrated and thus preferred a paper format.

## Discussion

### General Self-Efficacy

The main findings of this study were a statistically significant difference between the users and nonusers of MT4C with regard to general self-efficacy (the confidence in their ability to deal with difficult situations). General self-efficacy significantly increased in the user group.

Other studies have found that general self-efficacy has a significant positive relationship with the quality of life of family carers of people with chronic illness [28]. For family carers of persons with dementia, self-efficacy has been found to have a significant relationship with health outcomes such as mental health [29,30] and, in particular, is negatively correlated with depression [31,32]. In the larger study, hope was found to be positively associated with general self-efficacy [13]. When the hope of participants increased, so did their confidence in their ability to deal with difficult situations. However, in the larger study using the intent-to-treat analysis, which included nonusers, general self-efficacy was not found to be significantly different in the treatment versus the control group, but hope was. It was important to further examine differences between users and nonusers in the treatment group, as the finding that self-efficacy was higher in users versus nonusers supports the intervention model in which MT4C has the potential to increase quality of life by increasing self-efficacy.

### Reasons for Nonuse

The qualitative data suggested that caregiving demands, accessibility to the site, and preference for a paper version or face-to-face interaction were barriers to use for nonuser participants. Caregiving demands with subsequent family carer lack of energy and feelings of stress are consistent with the findings from the qualitative data from all participants in the larger study [19]. Quality of life scores at each time period for users and nonusers were not significantly different; however, whether the nonuser group experienced a more pronounced lack of energy and higher levels of stress than those in the user group is unclear. Future research should potentially also measure fatigue and stress as possible barriers to the use of Web-based interventions.

Poor connectivity to the internet was described as a barrier to use of MT4C by nonuser participants. Web-based interventions have been considered to be of benefit particularly for rural populations because of considerations related to accessibility [33]. However, poor connectivity to the internet, particularly for persons in rural areas in Canada, is a barrier to the use of any Web-based intervention [34,35]. Poor connectivity should be a concern for any research with Web-based interventions and possibly an exclusion criterion for participants in efficacy and effectiveness trials.

Computer literacy (ie, the ability to use computers and related technology efficiently) appeared to be one of the barriers to using MT4C. Inclusion criteria for the study included access to a computer and an email address. However, in this study, a measure of computer literacy was not used. Park et al [36], following an integrated review of health-related internet use of family carers of children, suggested that Web-based interventions should also include educational programs to increase computer literacy. Although MT4C was previously determined to be easy to use, an additional *tell me more* feature could be embedded into the program to assist carers who have low computer literacy.

What is unclear from our study is if access issues and computer literacy resulted in some participant preferences for a paper format and/ or face-to-face supportive interactions. Moreover, the nonusers referred in their comments to in-person interaction with other carers not Web-based interaction with other carers. When interaction with other family carers was added to a Web-based intervention, caregiving demand and computer literacy were also found to have an impact on the perceived benefit of a Web-based intervention [37]. This suggests that Web-based interventions to support family carers of persons with ADRD and MCC should not be the only format for support, but opportunities to use a paper version of MT4C and in-person face-to-face interactions are also important.

### Limitations

This study was a secondary analysis; thus, follow-up interviews with nonuser participants were not conducted. Follow-up interviews would have been completed to further explore participants’ reasons for nonuse and to answer questions about the relationship between computer literacy and their preferences for a paper format. Low computer literacy and poor connectivity were also not considered as exclusion criteria for the study, possibly influencing the results.

Importantly, this comparison involves a sample that was not randomly assigned, thus limiting the generalizability of the study. Although there were no statistically significant differences in the demographic characteristics between users and nonusers, there may be a potential imbalance between the groups based on unmeasured contextual characteristics. The findings contribute to the developing model of the intervention; however, in future studies, potential mediators and moderators should be identified and their influence on the outcomes of the intervention should be evaluated.

### Conclusions

The findings of this study reflect how comparisons of users and nonusers in Web-based intervention studies can improve Web-based interventions and the design of future studies. The statistically significant higher levels of general self-efficacy (or the confidence in the ability to deal with difficult situations) in users of MT4C is an important finding. Family carers of persons with dementia have reported significantly less self-efficacy than carers of persons without dementia [38]. As such, MT4C can potentially benefit family carers who are willing and able to use Web-based interventions. More research is needed to determine if adding an educational program for computer literacy may assist more family carers to access this Web-based intervention. In addition, future research should explore the use of MT4C in carers with diverse cultural backgrounds and languages.

## Abbreviations

ADRD: Alzheimer disease and related dementias
ANCOVA: analysis of covariance
GEE: generalized estimating equations
GSES: general self-efficacy scale
HHI: herth hope index
HRQOL: health-related quality of life
MCC: multiple chronic conditions
MCS: mental component score (SF-12v2)
MT4C: My Tools 4 Care
PCS: physical component score (SF-12v2)
SF-12v2: short-form 12-item health survey
